# Dipeptidyl peptidase 4 inhibitor improves insulin resistance in Japanese patients with type 2 diabetes: a single-arm study, a brief report

**DOI:** 10.1186/s13098-022-00850-9

**Published:** 2022-06-07

**Authors:** Tsuyoshi Okura, Yohei Fujioka, Risa Nakamura, Yuichi Ito, Sonoko Kitao, Mari Anno, Kazuhisa Matsumoto, Kyoko Shoji, Hiroko Okura, Kazuhiko Matsuzawa, Shoichiro Izawa, Etsuko Ueta, Masahiko Kato, Takeshi Imamura, Shin-ichi Taniguchi, Kazuhiro Yamamoto

**Affiliations:** 1 Division of Cardiovascular Medicine, Endocrinology and Metabolism, Faculty of Medicine, Tottori University, 36-1 Nishi-cho, Yonago, Tottori 683-8504 Japan; 2 School of Health Science, Faculty of Medicine, Tottori University, Yonago, Japan; 3 Division of Molecular Pharmacology, Faculty of Medicine, Tottori University, Yonago, Japan; 4 Department of Regional Medicine, Faculty of Medicine, Tottori University, Yonago, Japan

**Keywords:** Dipeptidyl peptidase 4 inhibitor, Insulin Resistance, Type 2 Diabetes, Glucose Clamp

## Abstract

**Background:**

Dipeptidyl peptidase 4 inhibitor (DPP4i) is an effective medicine for type 2 diabetes mellitus (T2DM). Some articles reported DPP4i improves insulin secretion and insulin resistance. However, these effects are not well established by glucose clamp test and test meal in Japanese. We investigated the effect of DPP4i on insulin resistance and insulin secretion by using the glucose clamp test and meal tolerance test (MTT).

**Methods:**

We performed a MTT, and the hyperinsulinemic-euglycemic clamp in 8 Japanese patients with T2DM. This study was a single-arm study. We measured fasting and postprandial glucose, insulin, incretins, and glucagon levels. We also measured serum adiponectin levels.

**Results:**

HbA1c was significantly decreased after 3 months. The fasting and postprandial glucose levels were significantly decreased. Fasting and postprandial insulin levels were not changed. The insulin resistance derived from the glucose clamp test was significantly improved. HOMA-IR was not significantly changed. GLP-1 and GIP were significantly increased but glucagon did not change. Adiponectin was not significantly changed.

**Conclusions:**

Although the number of patients was very small, these results suggested that DPP4i treatment might improve insulin resistance without changing insulin secretion.

## Introduction

The major pathophysiology of type 2 diabetes mellitus (T2DM) is increased insulin resistance and decreased insulin secretion [1]. Dipeptidyl dipeptidase inhibitor 4 inhibitors (DDP4i) are an anti-diabetic medicine, which increases incretin hormones, glucagon-like peptide-1 (GLP-1), glucose-dependent insulinotropic polypeptide (GIP), induce glucose-lowering effect [2]. DPP-4 inhibitors were firstly reported that improved glucose levels, increased insulin secretion, and improved beta-cell function in rodents [3]. However, a human study reported that DPP-4 inhibitor decreased fasting and postprandial plasma glucose, and HbA1c without changing fasting and postprandial insulin in T2DM patients [4]. Another study reported that sitagliptin increased post-load insulin and decreased post-load glucagon at 2 h in the oral glucose tolerance test (OGTT) compared with placebo [5]. We also previously reported sitagliptin improved postprandial glucose and early insulin response, however, the insulin secretion enhancing effects were very mild [6]. Previous another study reported that DPP4i improve insulin resistance evaluated by glucose clamp test in American, but insulin secretion did not change [7]. However, there are few reports about the effect of DPP4i on insulin resistance evaluated by the glucose clamp test in Asian and Japanese populations. East Asian and American show different pathophysiology of T2DM, East Asian shows insulin resistance with mild obesity and low insulin secretion ability [8]. Furthermore, there is a report that DPP4i increases serum adiponectin levels [9]. Adiponectin is important for insulin resistance in the Japanese population [10]. East Asians (Japanese, China, Korean, Taiwanese) showed a significantly greater effect of DPP4i compared with non-East Asians [11]. These results suggested that the effect of DPP4i on the East Asian population is different from other populations.

According to these results, DPP4i affects insulin resistance, however, the mechanisms are not well known in real-world settings, especially in the Asian and Japanese populations. Here, we show that DPP4i improves insulin resistance evaluated by the glucose clamp method in the Japanese population.

## Materials and methods

### Subjects

We recruited 8 Japanese patients with T2DM for this study at Tottori University Hospital from 2013 to 2014. T2DM was diagnosed under the World Health Organization (WHO) criteria [12]. We recruited the patients with age 20–80 years old, HbA1c 6.5-9.0, without oral hypoglycemic agents, and insulin injection. We excluded the patients with cancer, viral hepatitis, liver cirrhosis, pancreatitis, renal failure, or those who are taking medications that increase glucose levels such as corticosteroids. This study was a single-arm study design. The patients took DPP4i as follows; 3 patients with teneligliptin 20 mg, 3 with linagliptin 5 mg, 2 with alogliptin 25 mg. We performed the glucose clamp test and meal tolerance test before and 3 months after DPP4i treatment.

We performed this study according to the Declaration of Helsinki. The Ethics Committee of the Faculty of Medicine, Tottori University approved this study, approval number 2087, approval date 28/01/2013. The first patient was enrolled on 10 April, 2013. The clinical trial number was UMIN000011189, registered (14/07/2013), UMIN000046686, registered (21/01/2022)—Retrospectively registered. We obtained informed consent from all of the participants using a procedure approved by the Ethics Committee.

### Meal tolerance test (MTT)

We conducted MTT as we previously reported [6]. Briefly, the patients ate a test meal developed by the Japan Diabetes Society (460 kcal; 50% carbohydrate, 35% fat, and 15% protein). We measured plasma glucose, serum insulin at 0, 30, 60, 120, and 180 min after the test meal.

HbA1c was measured by using high-performance liquid chromatography (HPLC). We used HbA1c converter to convert from the percentage values to the mmol/mol values [13].

Insulin resistance and insulin secretion ability were calculated as follows:

- Homeostatic model assessment insulin resistance (HOMA-IR) [14] = [fasting plasma glucose (mmol/L)] × [fasting plasma insulin (pmol/L)]/135.
- Homeostasis model assessment of beta-cell function (HOMA-beta) [14] = {20 × [fasting plasma insulin (pmol/L)]}/{[fasting plasma glucose (mmol/L)] − 3.5}.
- Insulinogenic index (IGI) [15] = {[insulin (pmol/L) at 30 min] − [fasting plasma insulin (pmol/L)]}/{[glucose (mmol/L) at 30 min] − [fasting plasma glucose (mmol/L)]}.
- The area under the curve (AUC) was calculated by the horizontal method.

### GLP-1, GIP, and glucagon assays

We obtained blood samples at 0, 30, 60, and 120 min after the meal by using a DPP4 inhibitor-containing blood collection tube (BD™ P800, BD Japan, Tokyo, Japan). We extracted samples for incretin assay by ethanol and solid-phase method [16]. We measured intact GLP-1 using a GLP-1 (active) ELISA (Merck Millipore, Germany). We measured active GIP using the Human GIP, Active form Assay Kit (IBL, Japan). We measured glucagon levels using a glucagon ELISA (Mercodia, Sweden).

### Hyperinsulinemic–euglycemic clamp

We conducted the glucose clamp test 2 days after MTT as we previously reported [17]. Briefly, we used an artificial endocrine pancreas (STG 55; Nikkiso, Japan), and performed the hyperinsulinemic-euglycemic clamp to measure insulin sensitivity. We used the protocol with a priming insulin infusion at 100 mU/m^2^/min, and the plasma glucose level was maintained at 5.2 mmol/L (95 mg/dL). This insulin infusion protocol was reported as an insulin level of 1200 pmol/L at steady-state in T2DM patients [18]. The mean GIR during 90–120 min was defined as the glucose disposal rate (GDR), which mainly reflects peripheral insulin sensitivity.

### Body composition

Body composition was measured by using Tanita body composition analyzer (Tanita, Japan). We measured the percent body fat, body fat mass, lean body mass, skeletal muscle mass.

## Statistical analysis

We expressed data as the mean ± standard deviation. We used the Mann–Whitney *U* test to assess differences in the mean value of clinical parameters between before and after DPP4i treatment. Values of *p* < 0.05 were considered to be significant. We used PRISM8 software (GraphPad Software, San Diego, USA) for analyses.

We performed a power analysis to compare GDR before and after treatment as a primary endpoint by using an EZR calculator 23. The mean GDR before treatment was 4.30 ± 1.20, and the mean GDR after treatment was 6.24 ± 1.35, the difference was 1.94 ± 0.12, alpha = 0.05, 1-beta = 0.8, the sample size was 8 patients, the estimated power was 1.0.

## Results

**Fig. 1:**
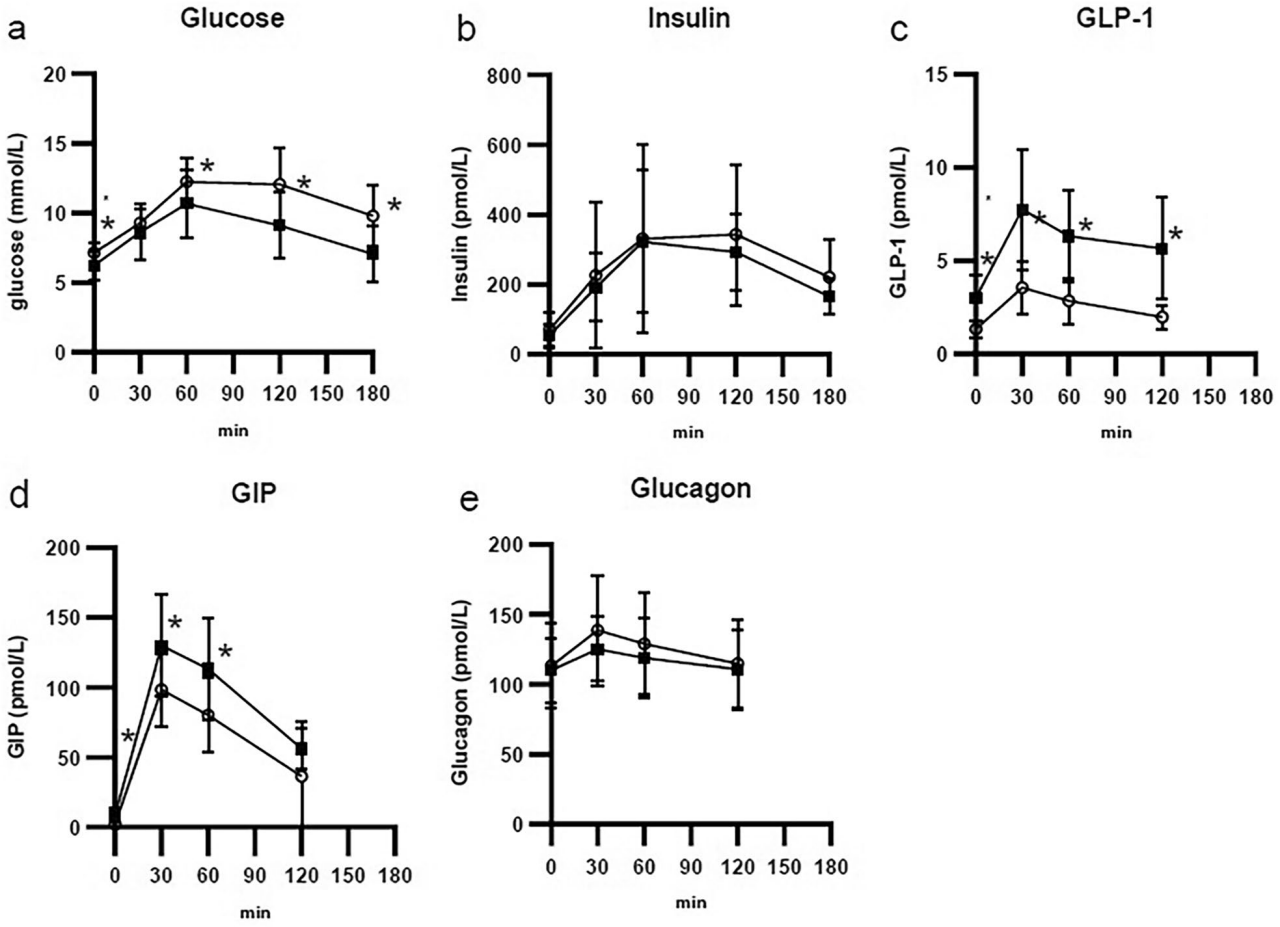
The meal tolerance test (MTT) results. **a** shows glucose levels after the MTT, **b** insulin, **c** GLP-1, **d** GIP, and **e** glucagon. White circles represent the data from pre-treatment, and black squares indicate post-treatment. Mann–Whitney *U* test. *p < 0.05

Participant characteristics are shown in Table 1. The mean age of patients was 59.6 ± 10.4 years old, and 6 males, 2 females. The duration of T2DM was 5.8 ± 6.0 years. Table 1 shows the effects of DPP4i after 3 months. Bodyweight and HbA1c were significantly changed after 3 months of treatment. Figure 1 shows MTT results. Fasting and postprandial 120 and 180 min glucose were significantly decreased. Insulin levels were not significantly changed. GLP-1 and GIP were significantly increased, but glucagon levels were not changed significantly. The insulin resistance of the glucose clamp was significantly improved, but HOMA-IR was not significantly changed (Table 1). All patients improved insulin resistance derived from the glucose clamp method (data not shown). HOMA-beta, insulinogenic index were not changed. Adiponectin was not changed. Body composition indicated that body fat mass was decreased.

**Table 1:** Comparison between before and after DPP4i treatment

Parameters	Before	After	p value
Body weight (kg)	70.0 ± 12.6	67.8 ± 12.5	< 0.05
BMI (kg/m^2^)	26.3 ± 4.8	25.3 ± 4.4	< 0.05
eGFR	65.9 ± 12.8	64.2 ± 13.8	NS
AST (U/L)	28.1 ± 12.7	20.5 ± 3.8	NS
ALT (U/L)	43.4 ± 25.9	23.0 ± 6.8	< 0.05
Gamma GTP (U/L)	46.1 ± 27.4	34.5 ± 11.7	NS
HbA1c (%)	7.8 ± 0.8	6.5 ± 0.7	< 0.005
HbA1c (mmol/mol)	61 ± 9	47 ± 8	< 0.005
Insulin secretion			
Fasting Plasma Glucose (mmol/L)	7.10 ± 0.70	6.22 ± 1.02	< 0.05
Glucose AUC (0–120 min)	21.6 ± 3.0	18.3 ± 4.1	< 0.05
Insulin AUC (0–120 min)	550.3 ± 387.7	496.7 ± 256.1	NS
HOMA-beta (%)	63.8 ± 42.2	79.8 ± 59.3	NS
Insulinogenic Index	1.00 ± 1.50	0.76 ± 0.68	NS
Insulin resistance			
HOMA-IR	3.8 ± 3.1	2.44 ± 1.19	NS
GDR (mg/kg/min)	4.30 ± 1.2	6.24 ± 1.35	< 0.01
Incretins			
GLP-1 AUC	5.2 ± 1.9	12.6 ± 4.3	< 0.001
GIP AUC	128.2 ± 47.1	180.1 ± 29.3	< 0.01
Glucagon AUC	251.9 ± 67.6	234.4 ± 51.3	NS
Adiponectin	5.0 ± 3.2	5.2 ± 3.2	NS
Body composition			
Percent body fat (%)	26.5 ± 9.0	24.4 ± 7.4	< 0.05
Body fat mass (kg)	18.8 ± 8.9	16.9 ± 7.4	< 0.05
Lean body mass (kg)	50.0 ± 8.8	49.8 ± 8.7	NS
Skeletal muscle mass (kg)	47.3 ± 8.4	47.2 ± 8.3	NS

Data are presented as the mean ± standard deviation

The comparison of parameters between before and after treatment was performed using the Mann–Whitney *U* test

Because the subjects showed significant weight reduction, we calculated GDR × BMI/22 to adjust GDR by BMI. The GDR × BMI/22 was significantly increased after DPP4i treatment (from 3.74 ± 1.38 to 5.73 ± 2.16, p < 0.001).

## Discussion

Our study showed that DPP4i improved the insulin resistance evaluated by the glucose clamp method. DPP4i decreased fasting and postprandial glucose levels without changing insulin levels. It is considered that HOMA-IR generally reflects hepatic insulin resistance, and GDR from glucose clamp reflects muscle insulin resistance [19]. The muscle insulin resistance derived from the glucose clamp method was improved by DPP4i. The HOMA-IR as a marker of liver insulin resistance was not significantly changed, but it was considered as tending to improvement. Previous studies showed DPP4i increased adiponectin [9], and we previously reported that adiponectin is important for the insulin resistance of Japanese [20]. However, Adiponectin was not changed. There is a report that GLP-1 improved muscle insulin resistance by vasodilatory actions [21]. Our study showed a significant increase of GLP-1, we consider GLP-1 is important for the improvement of muscle insulin resistance. Although further basic research of animals and cellular are needed for understanding the molecular mechanism, our results contribute to understanding the effects of DPP4i.

The previous study also showed DPP4i improves insulin resistance, but this study was performed in American [7]. East Asian and American show different pathophysiology of T2DM, East Asian shows low insulin secretion ability and insulin resistance with mild obesity [8, 22]. East Asians (Japanese, China, Korean, Taiwanese) showed a significantly greater effect of DPP4i compared with non-East Asians [11], our study might contribute to understanding one mechanism of DPP4i. All patients in our study are Japanese, therefore, our results contribute to the daily clinical work in the Japanese population.

Our study did not show a significant change in insulin secretion by DPP4i. Previous studies also did not show a significant change in insulin secretion [4, 7, 9]. A previous study reported that sitagliptin 25 mg and 200 mg increased insulin secretion [5], but this study was performed at 24 h DPP4i treatment, the short period might be different from the real-world situation. A recent large patient number Japanese study showed that DPP4i increased insulin levels, but the increase in insulin levels was very mild [23]. The previous study showed the improvement of insulin resistance without changing insulin levels [7], the authors described that these findings might be results of improvement of glucose toxicity. We also consider the effects of DPP4i on glucose toxicity is important. Moreover, there are some reports that DPP4i decreases the glucagon section from the alpha cell [24]. We did not see the significant glucagon inhibition by DPP4i, but it may depend on the number of small patients. Furthermore study is needed.

Our study has some limitations, the major limitation is a very small number of patients eight. However, the glucose clamp method and measuring incretins after a meal are very complicated and expensive studies, and it is difficult to recruit patients without any medication in the University hospital. We need a larger study. Our patients showed significant weight reduction. Generally, DPP4i does not change body weight [25], and our study was a single-arm study, we don’t have control patients, therefore, our study might have bias. However, the GDR adjusted by BMI also showed a significant increase, we consider DPP4i have the effect of improvement of insulin resistance. However, this BMI adjusting method might not reflect the true improvement of insulin resistance. We also suspect that body weight/fat reduction in three months directly improved insulin sensitivity, but DPP-4i indirectly through ameliorating glucotoxicity. If we would like to demonstrate the DPP-4i can directly improve insulin sensitivity, we should perform insulin clamp study in short term i.e. one month. Despite these limitations, we consider our study contributes to our daily clinical work.

In conclusion, although the number of patients was very small, these results suggested that DPP4i treatment might improve insulin resistance without changing insulin secretion.

## Data Availability

The datasets used and/or analysed during the current study available from the corresponding author on reasonable request. Data sharing statement: No additional data available.

## Abbreviations

AUC: Area under the curve
DPP4i: Dipeptidyl peptidase 4 inhibitor
eGFR: Estimate glomerular filtration rate
GDR: Glucose disposal rate
GIP: Glucose-dependent insulinotropic polypeptide
GLP-1: Glucagon-like peptide-1
HbA1c: Glycated hemoglobin
HOMA-beta: Homeostatic model assessment beta-cell function
HOMA-IR: Homeostasis model assessment for insulin resistance

## References

[1] Kahn SE, Cooper ME, Del Prato S (2014). Pathophysiology and treatment of type 2 diabetes: perspectives on the past, present, and future. Lancet.

[2] Ahren B, Schmitz O (2004). GLP-1 receptor agonists and DPP-4 inhibitors in the treatment of type 2 diabetes. Horm Metab Res.

[3] Ahren B, Holst JJ, Martensson H, Balkan B (2000). Improved glucose tolerance and insulin secretion by inhibition of dipeptidyl peptidase IV in mice. Eur J Pharmacol.

[4] Ahren B, Landin-Olsson M, Jansson PA, Svensson M, Holmes D, Schweizer A (2004). Inhibition of dipeptidyl peptidase-4 reduces glycemia, sustains insulin levels, and reduces glucagon levels in type 2 diabetes. J Clin Endocrinol Metab.

[5] Herman GA, Bergman A, Stevens C, Kotey P, Yi B, Zhao P, Dietrich B, Golor G, Schrodter A, Keymeulen B, Lasseter KC, Kipnes MS, Snyder K, Hilliard D, Tanen M, Cilissen C, De Smet M, de Lepeleire I, Van Dyck K, Wang AQ, Zeng W, Davies MJ, Tanaka W, Holst JJ, Deacon CF, Gottesdiener KM, Wagner JA (2006). Effect of single oral doses of sitagliptin, a dipeptidyl peptidase-4 inhibitor, on incretin and plasma glucose levels after an oral glucose tolerance test in patients with type 2 diabetes. J Clin Endocrinol Metab.

[6] Ohkura T, Fujioka Y, Sumi K, Nakanishi R, Shiochi H, Yamamoto N, Matsuzawa K, Izawa S, Ohkura H, Kato M, Taniguchi S, Yamamoto K (2014). Sitagliptin Improves the Impaired Acute Insulin Response during a Meal Tolerance Test in Japanese Patients with Type 2 Diabetes Mellitus: a Small-Scale Real-World Study. Diabetes Ther.

[7] Azuma K, Rádiková Z, Mancino J, Toledo FG, Thomas E, Kangani C, Dalla Man C, Cobelli C, Holst JJ, Deacon CF, He Y, Ligueros-Saylan M, Serra D, Foley JE, Kelley DE (2008). Measurements of islet function and glucose metabolism with the dipeptidyl peptidase 4 inhibitor vildagliptin in patients with type 2 diabetes. J Clin Endocrinol Metab.

[8] Hsu WC, Araneta MR, Kanaya AM, Chiang JL, Fujimoto W (2015). BMI cut points to identify at-risk Asian Americans for type 2 diabetes screening. Diabetes Care.

[9] Hibuse T, Maeda N, Kishida K, Kimura T, Minami T, Takeshita E, Hirata A, Nakagawa Y, Kashine S, Oka A, Hayashi M, Nishizawa H, Funahashi T, Shimomura I (2014). A pilot three-month sitagliptin treatment increases serum adiponectin level in Japanese patients with type 2 diabetes mellitus–a randomized controlled trial START-J study. Cardiovasc Diabetol.

[10] Kadowaki T, Sekikawa A, Okamura T, Takamiya T, Kashiwagi A, Zaky WR, Maegawa H, El-Saed A, Nakamura Y, Evans RW, Edmundowicz D, Kita Y, Kuller LH, Ueshima H (2006). Higher levels of adiponectin in American than in Japanese men despite obesity. Metabolism.

[11] Kim YG, Hahn S, Oh TJ, Kwak SH, Park KS, Cho YM (2013). Differences in the glucose-lowering efficacy of dipeptidyl peptidase-4 inhibitors between Asians and non-Asians: a systematic review and meta-analysis. Diabetologia.

[12] Alberti KG, Zimmet PZ (1998). Definition, diagnosis and classification of diabetes mellitus and its complications. Part 1: diagnosis and classification of diabetes mellitus provisional report of a WHO consultation. Diabet Med.

[13] National Institutes of Diabetes and Digestive and Kidney Diseases, the HbA1c converter. 1999. http://www.ngsp.org/convert1.asp.

[14] Matthews DR, Hosker JP, Rudenski AS, Naylor BA, Treacher DF, Turner RC (1985). Homeostasis model assessment: insulin resistance and beta-cell function from fasting plasma glucose and insulin concentrations in man. Diabetologia.

[15] Uwaifo GI, Fallon EM, Chin J, Elberg J, Parikh SJ, Yanovski JA (2002). Indices of insulin action, disposal, and secretion derived from fasting samples and clamps in normal glucose-tolerant black and white children. Diabetes Care.

[16] Yabe D, Eto T, Shiramoto M, Irie S, Murotani K, Seino Y, Kuwata H, Kurose T, Seino S, Ahrén B, Seino Y (2017). Effects of DPP-4 inhibitor linagliptin and GLP-1 receptor agonist liraglutide on physiological response to hypoglycaemia in Japanese subjects with type 2 diabetes: A randomized, open-label, 2-arm parallel comparative, exploratory trial. Diabetes Obes Metab.

[17] Okura T, Fujioka Y, Nakamura R, Anno M, Ito Y, Kitao S, Matsumoto K, Shoji K, Sumi K, Matsuzawa K, Izawa S, Okura H, Ueta E, Noma H, Kato M, Imamura T, Taniguchi SI, Yamamoto K (2020). Hepatic insulin clearance is increased in patients with high HbA1c type 2 diabetes: a preliminary report. BMJ Open Diabetes Res Care.

[18] Tamura Y, Tanaka Y, Sato F, Choi JB, Watada H, Niwa M, Kinoshita J, Ooka A, Kumashiro N, Igarashi Y, Kyogoku S, Maehara T, Kawasumi M, Hirose T, Kawamori R (2005). Effects of diet and exercise on muscle and liver intracellular lipid contents and insulin sensitivity in type 2 diabetic patients. J Clin Endocrinol Metab.

[19] Muniyappa R, Lee S, Chen H, Quon MJ (2008). Current approaches for assessing insulin sensitivity and resistance in vivo: advantages, limitations, and appropriate usage. Am J Physiol Endocrinol Metab.

[20] Ohkura T, Shiochi H, Fujioka Y, Sumi K, Yamamoto N, Matsuzawa K, Izawa S, Kinoshita H, Ohkura H, Kato M, Taniguchi S, Yamamoto K (2013). 20/(fasting C-peptide × fasting plasma glucose) is a simple and effective index of insulin resistance in patients with type 2 diabetes mellitus: a preliminary report. Cardiovasc Diabetol.

[21] Wang N, Tan AWK, Jahn LA, Hartline L, Patrie JT, Lin S, Barrett EJ, Aylor KW, Liu Z (2020). Vasodilatory Actions of Glucagon-Like Peptide 1 Are Preserved in Skeletal and Cardiac Muscle Microvasculature but Not in Conduit Artery in Obese Humans With Vascular Insulin Resistance. Diabetes Care.

[22] Okura T, Nakamura R, Fujioka Y, Kawamoto-Kitao S, Ito Y, Matsumoto K, Shoji K, Sumi K, Matsuzawa K, Izawa S, Ueta E, Kato M, Imamura T, Taniguchi SI, Yamamoto K (2018). Body mass index ≥23 is a risk factor for insulin resistance and diabetes in Japanese people: A brief report. PLoS ONE.

[23] Abe T, Matsubayashi Y, Muragishi S, Yoshida A, Suganami H, Furusawa K, Fujihara K, Tanaka S, Kaku K, Sone H (2021). The DPP-4 inhibitor, anagliptin, alters hepatic insulin clearance in relation to the glycemic status in Japanese individuals with type 2 diabetes. J Diabetes Investig.

[24] Baranov O, Kahle M, Deacon CF, Holst JJ, Nauck MA (2016). Feedback suppression of meal-induced glucagon-like peptide-1 (GLP-1) secretion mediated through elevations in intact GLP-1 caused by dipeptidyl peptidase-4 inhibition: a randomized, prospective comparison of sitagliptin and vildagliptin treatment. Diabetes Obes Metab.

[25] Tsapas A, Karagiannis T, Kakotrichi P, Avgerinos I, Mantsiou C, Tousinas G, Manolopoulos A, Liakos A, Malandris K, Matthews DR, Bekiari E (2021). Comparative efficacy of glucose-lowering medications on body weight and blood pressure in patients with type 2 diabetes: a systematic review and network meta-analysis. Diabetes Obes Metab.

